# Reliability and validity of Hindi translation of the migraine disability assessment and headache impact test-6 questionnaires

**DOI:** 10.4103/0972-2327.74201

**Published:** 2010

**Authors:** Ratish Juyal, Rajesh Verma, Ravindra Kumar Garg, Rakesh Shukla, Atul Agarwal, Maneesh Kumar Singh

**Affiliations:** Department of Neurology, Chhatrapati Shahuji Maharaj Medical University, Uttar Pradesh, Lucknow, India

**Keywords:** Headache Disability, HIT-6, migraine disability assessment, migraine, reliability, validity

## Abstract

**Objective:**

The objective of the study was to assess the reliability and validity of the Hindi translation of the Migraine Disability Assessment (MIDAS) and Headache Impact Test-6 (HIT-6) questionnaires.

**Materials and Methods:**

The study was conducted on the migraine patients. For test–retest reliability, the respondents filled the MIDAS and HIT-6 questionnaires twice, at an interval of three weeks. For validity, the same population of patients filled the headache diary for three months. After three months they filled the MIDAS and HIT-6 questionnaires again. The patients were subgrouped according to their occupation and level of education. The test–retest reliability and validity were calculated by the Pearson correlation coefficient. Internal consistency was calculated using the Cronbach alpha.

**Results:**

A total of 236 migraine patients were screened. Seventy-nine patients fulfilled the inclusion criteria. A total of 69 patients completed the study. The HIT-6 questionnaire was applicable to all the subgroups of patients and had better comprehensibility than the MIDAS. Housewives missed out on the first two questions of the MIDAS and had lower mean MIDAS scores than HIT-6. The test–retest correlation coefficients for the total MIDAS and HIT-6 scores were 0.94 and 0.81, respectively. The correlation coefficients between the total score in the headache diary equivalent and the MIDAS and HIT-6 total score were 0.91 and 0.77, respectively. Cronbach alpha, a measure of internal consistency for the MIDAS questionnaire was > 0.90 at all the compilations. For the HIT-6 questionnaire, it ranged from 0.67 to 0.79.

**Conclusion:**

The Hindi versions of MIDAS and HIT-6 questionnaires were reliable and valid, but could not be interchanged. HIT-6 had better comprehensibility.

## Introduction

The Migraine disability assessment questionnaire (MIDAS) and Headache impact test (HIT-6) are two widely used self-administered questionnaires to assess headache-related disability. The self-administered disability questionnaire serves as a screening tool to identify people in need of urgent medical care, helps in improving patient–doctor communication, and provides an outcome measure for clinical practice, clinical trials, and epidemiological research.[1]

The MIDAS is a seven item questionnaire developed to measure headache disability. It measures disability in three domains of daily activity during the past three months. Items 1 to 5 measure disability in relation to these three domains, namely paid work or school work, household work, and leisure. Two items (A and B) enquire about the number of headache days in the last three months and the severity of the headache on a scale ranging from 0 to10, respectively. Only items 1 to 5 are added to the total score. The reliability and the validity of English, Japanese, Turkish, and Chinese (Taiwan) versions of the MIDAS questionnaire have been established.[2–7] The test–retest reliability has also been established in French and Italian.[8,9]

The Headache Impact Test (HIT-6) was developed in 2003.[10] It is different from the MIDAS questionnaire in terms of the period of headache recall, which is only four weeks in the HIT-6 questionnaire. It has six questions. Each question has five options and the respondents have to encircle one of the options. It has been translated in 27 countries.[11]

The reliability and validity of MIDAS and HIT-6 questionnaires have not been tested in Hindi, the national language of India. It has to be seen whether these questionnaires are applicable to the culturally different and diverse Indian population. The objective of the study is to assess the reliability and the validity of the Hindi translation of MIDAS and HIT-6 questionnaires.

## Materials and Methods

**Figure 1:**
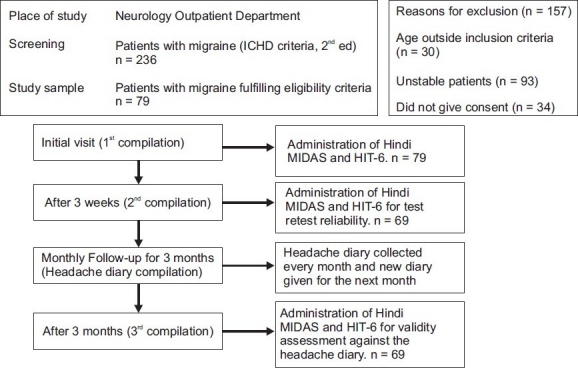
Plan of study

The study was conducted in the Neurology Outpatient Department of the CSM Medical University, Lucknow, a large tertiary hospital in North India. The duration of the study was one year. The plan of the study is depicted in Figure 1. The diagnosis of migraine was based on the revised International Headache Classification (ICHD) criteria, second edition.[12] The study was approved by the institutional Ethics Committee and a written informed consent was taken prior to enrollment. Patients between 18 to 55 years of age, suffering from migraine for at least three months, and stable, so that no change in migraine medication was required, were enrolled. A predesigned structured proforma was used to record the demographic characteristics as well as the headache history.

### Translation of the questionnaires into Hindi

**Figure 2:**
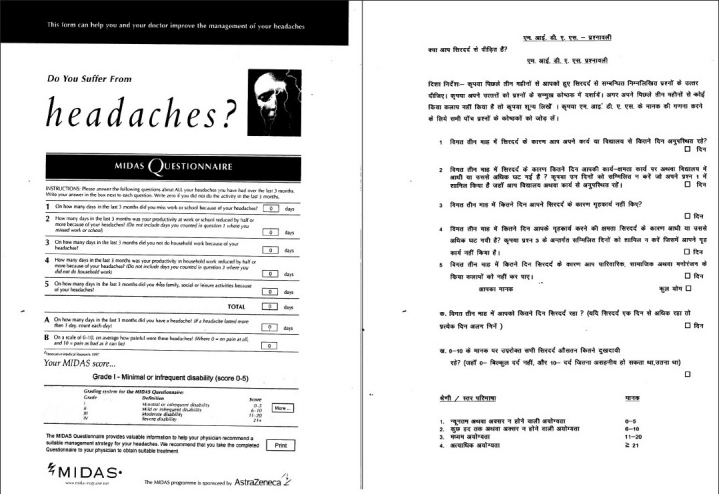
MIDAS – hindi version

**Figure 3:**
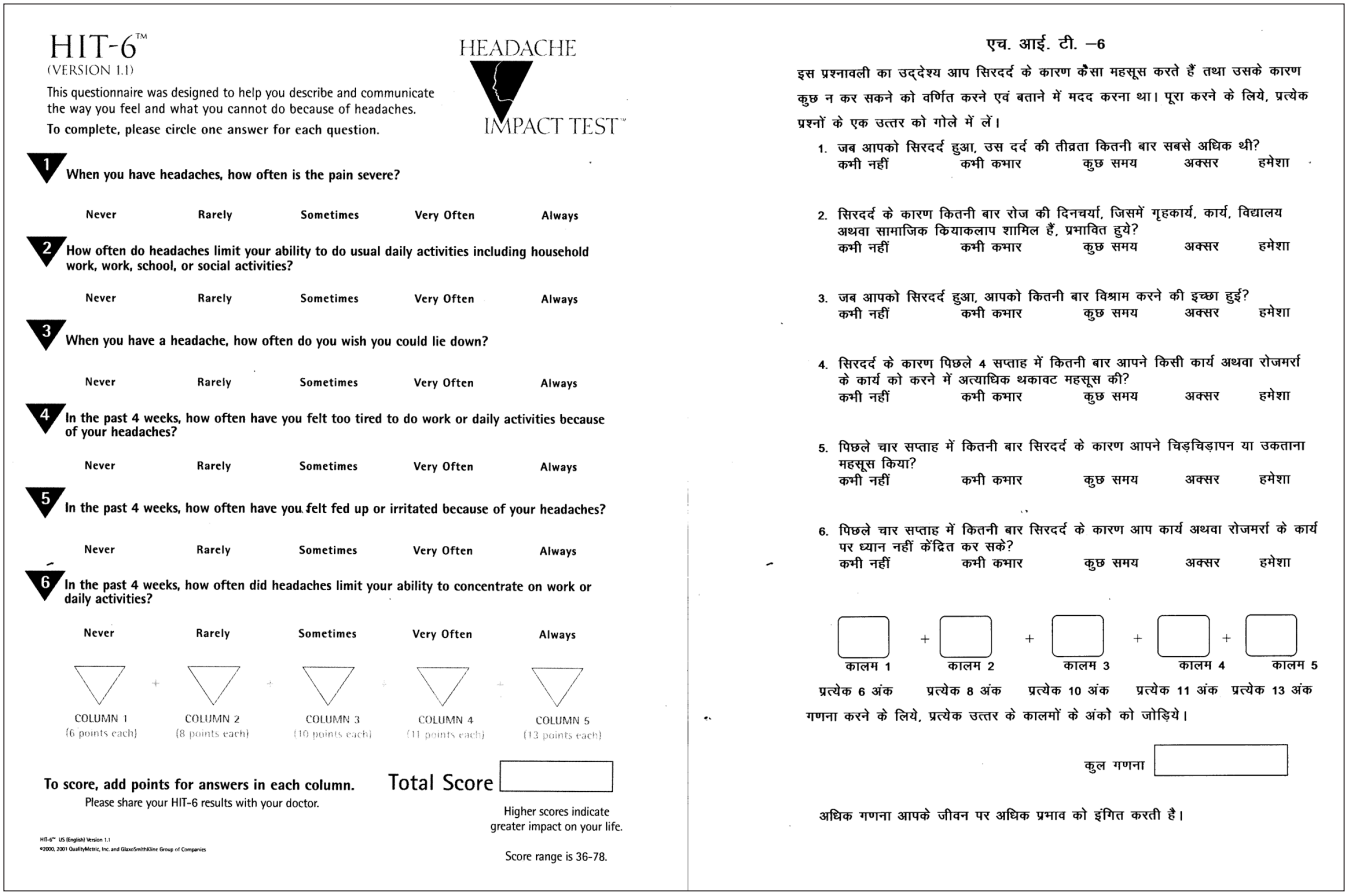
HIT-6 – hindi version

The Migraine disability assessment and HIT-6 questionnaires were translated into Hindi by the standard process of translation and back translation by two independent translators [Figures 2 and 3]. One of them translated questionnaires into Hindi; and the other translated Hindi versions back into English to check the comparability with the original English version.

### First compilation of the questionnaires

The patients completed the Hindi versions of MIDAS and HIT-6 questionnaires [Figures 2, 3] during the initial visit. They were given a headache diary and explained to fill it daily at the same time each day, preferably at bed time. The headache diary consisted of two parts. The first part was to be filled daily. It contained general questions regarding daily activities and sense of well being or mood of the patient. The second part was to be filled during the headache days and questions were asked about the headache characteristics and its impact. Each item in MIDAS and HIT-6 questionnaires was incorporated in the headache diary. The purpose of giving the headache diary during the initial visit was to ensure that patients were adequately trained to fill the diary before starting a validation study. For illiterate patients (n = 10), questions contained in the questionnaires were read out by investigators. Such patients were asked to get the headache diary filled daily and on the headache days with the help of any literate person easily accessible to them. If this was not possible such patients were excluded from the study.

### Second compilation of the questionnaires for test–retest reliability

The first follow up was after three weeks. This time interval was the same as used in the original MIDAS study and was considered to be short enough to eliminate any significant change in headache severity, but long enough to prevent the patients from recalling the responses made in the previous questionnaires.[2] Patients were telephonically reminded about the first follow-up a week in advance, wherever it was possible. The MIDAS and the HIT-6 questionnaires were filled a second time. The headache diary was checked for any mistake. Patients were asked for any difficulty with the headache diary and if any difficulty was there, it was sorted out by the investigators.

### Headache diary and third compilation of the questionnaires for validity testing

The same patients who participated in the test–retest reliability were followed up at intervals of one month for three months. The patients were asked to bring the headache diary during each follow-up. A new headache diary for the next month was given to the patient. At the end of three months, the patients completed the MIDAS and HIT-6 questionnaires for the third time.

### Statistical analysis

The sample size was calculated to be 45 patients using 90% power of study, with 0.60 as the correlation coefficient and level of significance at 5%. The mean values of the total scores as well as individual items were also calculated. Test–retest reliability was analyzed for the total scores as well as for the individual item scores obtained from the first and the second compilations of the MIDAS and the HIT-6 questionnaires, by calculating the Pearson correlation coefficient (r).The patients were also subgrouped according to their occupation (working, housewives, and students) and level of education (illiterate, below high school, and above high school). Internal consistency was measured using the Cronbach alpha. Validity was tested by calculating the Pearson correlation coefficient between the retrospective headache data obtained from the third compilations of the MIDAS and HIT-6 questionnaires with its equivalent, prospective data, collected every month for three months from the headache diary. A *P* value less than .05 was considered significant. Patients who lost to follow-up (n = 10), were not included in the data analysis.

## Results

A total number of 236 migraine patients were screened. Out of 236 patients, 79 patients fulfilled the inclusion criteria and enrolled in the study. Sixty-nine patients completed the study [Table 1]. Ten (12.6%) patients did not turn up after the first compilation of MIDAS and HIT-6 questionnaires. In the study, 47 patients resided in the urban area and 22 patients were from the rural background.

**Table 1:** Demographic characteristics

Variable	No.	%
Age		
<20	15	21.7
21 – 30	27	39.1
31 – 40	14	20.3
41 – 50	11	15.9
51 – 60	2	2.9
Gender		
Female	48	69.6
Male	21	30.4
Migraine type		
Migraine without aura		
Episodic	48	69.6
Chronic	14	20.3
Migraine with aura		
Episodic	6	8.7
Chronic	1	1.4
Mean duration of headache (Years)	7.82 ± 3.45	
Education		
Illiterate	10	14.5
Below high school	9	13.0
High school and above	50	72.5
Occupation		
Housewife	22	31.9
Student	19	27.5
Working	28	40.6
Office goers	8	
Businessmen	14	
Farmers	6	

### Comprehensibility of the questionnaires

Overall, the comprehensibility of the MIDAS was good. Fifty-two patients (75.3%) had difficulty in understanding item B of the MIDAS and had to be helped while filling the questionnaire at the first compilation. Two patients (2.8%) needed to be explained the difference between missed days and more than 50% reduction in the productivity in various domains of daily activity.

For the MIDAS questionnaire all the subgroups filled every item except housewives, who missed out on item 1 and item 2, where the number of days missed or more than 50% reduction in productivity at work or school were asked, respectively. The reason for not excluding housewives from the data analysis was that they accounted for a considerable percentage of patients (31.2%, n = 22). Therefore, mean for item 1 and item 2 could be calculated only for 47 patients after excluding housewives. For the rest of the items and total scores, the mean was calculated for all the 69 patients. Similarly test–retest correlation coefficient for item 1 and item 2 was calculated for 47 patients only, as the housewives did not fill responses to these items. Test–retest correlation coefficient for the rest of the items and total scores was calculated for all the patients (n = 69), as responses to these were filled by every patient. The internal consistency was possible only after excluding the housewives as they missed out on item 1 and 2 of the MIDAS questionnaire. For illiterate patients (n = 10) the questionnaire was administered by the physician at all visits.

HIT-6 questionnaire had excellent comprehensibility and none of the patients had difficulty in filling the questionnaire. HIT-6 was uniformly applicable to all the participants and all the items were answered by every participant. Both the questionnaires were well comprehended by the patients irrespective of their rural or urban background. There was no difference between the urban and rural subgroups with regard to the reliability and validity of these questionnaires.

### MIDAS and HIT-6 Scores

The mean of the total and individual item scores in different compilations of questionnaires in all patients as well as after dividing them into occupational and education-wise subgroups is shown in Tables 2, 3, and 4.

**Table 2:** MIDAS and HIT-6 scores at different compilations

MIDAS	n	First compilation			Second compilation			Third compilation		
		Mean	SD	Median	Mean	SD	Median	Mean	SD	Median
1	47	5.72	6.68	3	7.00	7.36	4	6.21	6.51	3
2	47	8.23	9.38	4	7.02	6.92	4	8.51	9.33	4
3	69	6.71	6.32	4	7.12	6.94	4	6.51	6.13	4
4	69	9.22	8.96	5	8.59	8.31	5	8.54	8.43	5
5	69	7.30	8.33	5	7.51	8.76	4	7.80	8.33	5
Total	69	32.72	32.53	21	32.77	31.23	20	32.48	31.67	19
A	69	15.33	14.03	10	16.67	14.41	12	15.57	13.32	10
B	69	8.81	1.37	9	8.49	1.44	8	8.83	1.42	10
**HIT-6**										
Total Score	69	60.61	7.26	59.00	59.29	6.21	60.00	60.54	7.20	60.0

MIDAS = Migraine disability assessment scale, HIT-6 = Headache impact test-6

**Table 3:** Occupation-wise Comparison of MIDAS and HIT-6 Scores

Compilations	Housewives (n = 22)		Students and Working (n = 47)		Statistical significance	
	Mean	SD	Mean	SD	t	*P*
MIDAS						
1	27.05	21.02	35.38	36.60	0.992	0.325
2	28.95	25.28	34.55	33.75	0.691	0.492
3	26.55	21.93	35.26	35.19	1.066	0.290
HIT-6						
1	62.86	6.58	60.23	10.10	1.113	0.270
2	61.68	5.77	58.17	6.15	2.253	0.028
3	63.45	6.95	50.17	6.96	2.383	0.020

MIDAS = Migraine disability assessment scale, HIT-6 = Headache impact test-6

**Table 4:** Education-wise comparison of MIDAS and HIT-6 Scores

Compilation	Illiterate (n=10)		Literate (n=59)		Statistical Significance		Below HS (n = 9)		Above HS (n = 59)		Statistical Significance	
	Mean	SD	Mean	SD	t	*P*	Mean	SD	Mean	SD	t	*P*
MIDAS												
1	31.80	37.50	32.88	31.97	0.096	0.923	59.80	6.07	61.29	9.61	0.472	0.638
2	27.60	23.69	33.64	32.41	0.563	0.575	58.80	5.79	59.37	6.32	0.268	0.790
3	24.70	23.03	33.80	32.89	0.838	0.405	59.40	5.76	60.73	7.44	0.537	0.593
HIT-6												
1	59.80	6.07	61.29	9.61	0.472	0.638	63.69	8.41	59.91	7.07	1.631	0.108
2	58.80	5.79	59.37	6.32	0.268	0.790	62.23	6.15	58.57	6.20	1.885	0.064
3	59.40	5.76	60.73	7.44	0.537	0.593	63.15	7.58	60.04	7.33	1.341	0.185

HS = High school

### Grade stratification and change

During the first compilation 79.7% patients were in MIDAS grades 3 and 4, and 84% patients had high HIT-6 scores (56 and above). At the second compilation 81.1% patients were in MIDAS grades 3 and 4, and 78.3% patients had high HIT-6 scores (56 and above). There was no significant change or shift in the grades between the first and second compilations for both the questionnaires.

### Test–retest reliability and internal consistency

For test–retest reliability, the participants completed the MIDAS and HIT-6 questionnaires twice; first at the initial visit and then after three weeks (n = 69). Test–retest correlation coefficients for the total scores as well as for the individual items are shown in Table 5. Test–retest correlation coefficients were calculated for: (1) All the patients, (2) after excluding housewives, (3) after excluding illiterate patients, and (4) after excluding both housewives and illiterate patients. For the MIDAS, correlation coefficients between the first and second compilations for all items (except for Item B) as well as for total scores were strong (r > 0.80). For item B, it was weak. The trend was similar in all four groups. The correlation between the total HIT-6 score at the first and second compilations was also strong in the first three groups of patients. However, for the fourth group (after excluding both housewives and illiterate patients), it was moderate(r = 0.64).

**Table 5:** Test–retest reliability

	All the Patients			Excluding HW			Excluding IL			Excluding HW and IL		
	n	r	*P*	n	r	*P*	n	r	*P*	n	r	*P*
MIDAS Item												
1	47	0.82	<0.001	47	0.82	<0.001	59	0.83	<0.001	44	0.83	<0.001
2	47	0.93	<0.001	47	0.93	<0.001	59	.0.93	<0.001	44	0.93	<0.001
3	69	0.85	<0.001	47	0.89	<0.001	59	0.85	<0.001	44	0.90	<0.001
4	69	0.87	<0.001	47	0.92	<0.001	59	0.87	<0.001	44	0.91	<0.001
5	69	0.87	<0.001	47	0.89	<0.001	59	0.89	<0.001	44	0.89	<0.001
Total	69	0.94	<0.001	47	0.95	<0.001	59	0.94	<0.001	44	0.96	<0.001
A	69	0.93	<0.001	47	0.95	<0.001	59	0.93	<0.001	44	0.95	<0.001
B	69	0.43	<0.001	47	0.43	0.002	59	0.42	<0.001	44	0.42	0.002
**HIT-6**												
Total	69	0.81	<0.001	47	0.88	<0.001	59	0.81	<0.001	44	0.64	< 0.001

HW = Housewives, IL = Illiterate patients, r = Pearson correlation coefficient

The internal consistency of the MIDAS score was high (Cronbach α > 0.9) at all the three compilations. For the HIT-6 score, the internal consistency was high (Cronbach α > 0.7) at the first and third compilations. At the second compilation, it was moderately acceptable (Cronbach α = 0.67).

### Validation of the MIDAS and HIT-6 questionnaires

Both MIDAS and HIT-6 total scores correlated well with the headache diary equivalent. The correlation was 0.91for MIDAS and 0.77 for HIT-6 [Table 6].

**Table 6:** Validation of MIDAS and HIT-6 with Headache Diary Equivalent

MIDAS		Compilation 3			HDE			n	r	*P*
	n	Mean	SD	Median	Mean	SD	Median			
Item 1	47	6.21	6.51	3	5.32	5.90	4	47	0.85	<0.001
Item 2	47	8.51	9.33	4	8.26	9.08	5	47	0.88	<0.001
Item 3	69	6.51	6.13	4	5.59	6.85	4	69	0.75	<0.001
Item 4	69	8.54	8.43	5	8.94	10.16	6	69	0.83	<0.001
Item 5	69	7.80	8.33	5	8.10	9.97	5	69	0.87	<0.001
Total	69	32.48	31.67	19	31.91	33.22	20	69	0.91	<0.001
A	69	15.57	13.32	10	15.55	14.28	10	69	0.91	<0.001
B	69	8.83	1.42	10	8.51	1.52	8	69	0.42	<0.001
**HIT-6**										
Total Score	69	60.54	7.20	60.0	57.57	6.40	58.00	69	0.771	0.001

HDE = Headache diary equivalent, r = Pearson correlation coefficient

### Correlation between MIDAS and HIT-6

Overall, a moderate-to-weak correlation (r = 0.64 to 0.48) was seen between MIDAS and HIT-6 scores, at all the three compilations [Table 7].

**Table 7:** Correlation between MIDAS and HIT-6 Questionnaire

Compilation No.	Total (n = 69)		
	n	r	*P*
1	69	0.64	<0.001
2	69	0.54	<0.001
3	69	0.48	<0.001

## Discussion

The mean age of the patients in our study was 30.07 years. Nearly 65% of the patients were between 20 and 50 years of age. In a tertiary hospital-based study in India, females formed 72% of the total migraine patients.[13] In our study, females constituted 69.6% of the total patients.

### Comprehensibility of the questionnaires

Overall, the comprehensibility of MIDAS was good, but a large number of respondents had difficulty in filling item B, where the patients were asked to grade severity of pain on a scale ranging from 0 to 10. Item B of MIDAS required a basic understanding of scales, which could not be expected from the general population of our country, where the literacy rate is not high.[14] In our population, it was recommended that instead of using a point scale, the adaptation of a more simple scale, like a rupee scale, would be more fruitful. In the Turkish MIDAS study, the patients were given a comprehension assessment form. Out of 107 patients, 65.7, 77.5, and 82% reported that they fully understood the questionnaire on visits 1, 2, and 3, respectively.[6] In the French version of MIDAS, the patients had difficulty with item A of MIDAS, and had a tendency to fill a range instead of the exact number of days.[8]

Our society is different from the western society. Housewives are expected to do the household chores and hence do not work outside. While filling the MIDAS questionnaire, housewives missed out on items 1 and 2, where the number of days missed or more than 50% reduction in productivity at work or school are asked, respectively. The reason for not excluding them from the data analysis is that they accounted for a considerable percentage (31.2%) of patients. In a study done in Taiwan, housewives constituted 17.3% of the total migraine patients. The details of how they fared on items 1 and 2 of the MIDAS as well as other items were not given in the study by the authors.[7]

HIT-6 was uniformly applicable to all the participants, and all the items were answered by every participant. HIT-6 had excellent comprehensibility and the respondents did not ask for any help. The reason that could be attributed to this difference in the comprehension of the two questionnaires by respondents could be the nature of the two questionnaires. HIT-6 was a qualitative questionnaire and items had options on a relative scale, which was easier to fill, while the MIDAS questionnaire required answers in exact numbers.

### Scores and grade stratification

The average MIDAS scores at all the three compilations was nearly 32. However, the range varied from 4 to 133 in different compilations. This wide range was due to the inability of the MIDAS questionnaire to record the housewives’ responses to items 1 and 2. The maximum score was contributed by item 4, which assessed loss of productivity in household work. This was in contrast to studies carried out in some countries, such as, U.K, France, Taiwan, and Italy, where the maximum score was related to loss of productivity in paid work, that is, item 2. Housewives accounted for 31.2% of our patients. This social difference could be responsible for the higher points given to the household work.[2,7–9]

The average HIT-6 score in every compilation was also consistent (nearly 60), similar to the MIDAS score. The range was from 38 to 78. The difference in range of scores for MIDAS (4 to 133) and HIT-6 (38 to 78) was due to the inability of the MIDAS scale to record housewives’ response on items 1 and 2.

The mean scores of the students and working group were higher when compared with those of the housewives on all the three compilations of MIDAS, although it was not statistically significant [Table 3]. In contrast, in the HIT-6 compilations, the mean scores of housewives were higher as compared to students and working groups, although the difference between the two groups was statistically significant in the second and third compilations. The reason for a lower mean score in MIDAS was due to housewives missing out on two items in the MIDAS questionnaire, unlike other occupational groups. Housewives filled all the items in the HIT-6 questionnaire as any other occupational group. We believe that MIDAS underestimates the severity of headache in housewives.

The MIDAS and HIT-6 scores did not depend upon the education level, and the disability was estimated irrespective of the education status, as the difference in the mean of total MIDAS and HIT-6 scores in illiterate patients, literates or various education groups was not statistically significant [Table 4]. There were two illiterate patients in the Turkish MIDAS study. The information regarding the methodology of filling the questionnaire by illiterate patients and their scoring pattern could not be gathered from the study.[6]

The majority of patients was in higher grades (MIDAS grade 3 and 4, HIT-6 score 56 or above), which reflects severe disability due to migraine. There were no significant changes in the grades between the first and second compilation.

### Test–retest reliability and internal consistency

Test–retest reliability for the total MIDAS score (r = 0.94) was higher than any other study [Table 8]. Reliability of the individual items 1 – 5 and item A, although not as high as the total score, was strong (r > 0.8). One of the reasons for this strong correlation could be the fact that our study included only those patients in whom the frequency of the headache (number of headaches in a month) was not showing large fluctuations. They were on migraine prophylaxis and took medications for acute attacks whenever needed. No attempt was made to change the previous treatment. The test–retest reliability of item B was weak. Item B, as discussed earlier, had poor comprehensibility in our patients. This may account for poor reproducibility at the second compilation. Test–retest reliability after excluding housewives, illiterate patients or both, showed a trend similar to the overall migraine patients. This suggested that housewives, despite missing out on two items, and illiterate patients in whom the questionnaire was administered by the investigators, did not affect the test–retest reliability in the overall population.

**Table 8:** MIDAS Test–retest reliability in various languages

Country	India*	USA	UK	Italy	Japan	Turkey	Taiwan	France
Year	2009	1999	1999	2001	2003	2004	2006	2007
Patients	69	97	100	109	99	107	31	143
Study	Hospital-based	Population-based	Population-based	Hospital-based	Hospital-based	Hospital-based	Hospital-based	Hospital-based
r	*P* 0.94	*P* 0.80	*P* 0.83	*P* 0.81	S 0.83	S 0.68	S 0.67	Sf 0.84

* Present study, r = correlation coefficient, *P* = Pearson, S = Spearman, Sf = Shrout- Fleiss

The test–retest reliability (r = 0.81) of the HIT-6 was comparable to its English version.[10]

Internal consistency ranged from moderate-to-strong and was slightly on the lower side as compared to the English version of HIT-6.The test–retest reliability was strong after excluding housewives and illiterate patients. It was moderate after excluding both the subgroups.

### Validation of MIDAS and HIT-6 questionnaires

The MIDAS questionnaire showed excellent validity (r = 0.91). Correlation between MIDAS (third compilation) and the headache diary equivalent was good for the total score as well as for the individual items except for item B, which was weakly correlated. The correlation in the English and Japanese version, in which the validity was calculated by the headache diary, was 0.63 and 0.66, respectively.[4,5] In our study, patients who participated in the validity and reliability studies were the same. The respondents had already filled the questionnaires twice in the reliability study, so they had a good idea about the items that were asked after three months of the validity assessment. This may be the reason for the strong correlation between the questionnaires and the headache diary equivalent.

HIT-6 had acceptable validity and the correlation between the total HIT-6 score and headache diary equivalent was 0.77. In the original HIT-6 study, validity in discriminating across diagnostic and headache severity groups and relative validity coefficients of 0.82 and 1.00 were observed for HIT-6, in comparison with the total score.[10]

### Correlation between MIDAS and HIT-6

In our study, the correlation between MIDAS and HIT-6 was weak. The MIDAS questionnaire being quantitative was different from the HIT-6, which was a qualitative questionnaire. As suggested in an earlier study, although both measured the disability due to headache, they could not be interchanged. In a recent study, a positive correlation was found between HIT-6 and MIDAS scores. The authors of the study concluded that the HIT-6 and MIDAS measured headache-related disability in a similar fashion. However, the headache intensity appeared to influence the HIT-6 score more than the MIDAS, whereas, the MIDAS was influenced more by headache frequency. Using the HIT-6 and MIDAS together was advocated to give a more accurate assessment of a patient’s headache-related disability.[15]

### Limitations of the study

In our study, the samples for reliability and validity testing were the same. The respondents filled the questionnaires twice in the reliability study. The same respondents participated in the validation study and were given monthly headache diaries for three months. After three months, the respondents filled the questionnaires for the third time in the validity assessment against the headache diary. As the patients had already filled the questionnaires twice, they had good idea about the questionnaires. This might have resulted in the strong correlation between the questionnaires and the headache diary equivalent in the validation study. For assessing the validity, it would have been more appropriate if we had included migraine patients who were not aware of the questionnaires. Our study was also limited by the fact that a good number of the patients, who missed out on the first two items of the MIDAS, were included in the study. The reason for their inclusion has already been discussed. Although MIDAS and HIT-6 were self-administered questionnaires, illiterate patients filled the questionnaires with the help of the investigators. This might have caused some bias. Nevertheless, through this study we tried to reach out to all the population subgroups.

## Conclusion

Overall, the comprehension of Hindi translations of MIDAS and HIT-6 questionnaires is good. We feel that instead of using a point scale, the adaptation of a more simple scale, such as a rupee scale, will be more fruitful in our population for item B of the MIDAS questionnaire. Both questionnaires are reliable and valid, but cannot be interchanged due to a weak-to-moderate correlation between them. In general, HIT-6 has acceptable reliability and validity, and is applicable to diverse population groups. MIDAS in present form, although it has strong reliability and validity, is not applicable to subgroups like housewives, who missed out on the first two items of the questionnaire. It underestimates the disability in housewives. Item 1 and item 2 of the Hindi version of MIDAS needs some modification to suit housewives, who form a major portion of migraine patients in our population. The study underscores the importance of keeping in mind the social structure of the region while developing a disability scale or the quality of life questionnaire.
